# Free-Form-Fabricated Commercially Pure Ti and Ti6Al4V Porous Scaffolds Support the Growth of Human Embryonic Stem Cell-Derived Mesodermal Progenitors

**DOI:** 10.1100/2012/646417

**Published:** 2012-01-04

**Authors:** G. M. de Peppo, A. Palmquist, P. Borchardt, M. Lennerås, J. Hyllner, A. Snis, J. Lausmaa, P. Thomsen, C. Karlsson

**Affiliations:** ^1^Department of Biomaterials, Sahlgrenska Academy at University of Gothenburg, P.O. Box 412, 413 46 Göteborg, Sweden; ^2^BIOMATCELL VINN Excellence Center of Biomaterials and Cell Therapy, 413 46 Göteborg, Sweden; ^3^SP Technical Research Institute of Sweden, 501 15 Borås, Sweden; ^4^Research and Development Division, Cellartis AB, 413 46 Göteborg, Sweden; ^5^Research and Development Division, Arcam AB, 431 37 Göteborg, Sweden

## Abstract

Commercially-pure titanium (cp-Ti) and the titanium-aluminum-vanadium alloy (Ti6Al4V) are widely used as reconstructive implants for skeletal engineering applications, due to their good mechanical properties, biocompatibility and ability to integrate with the surrounding bone. Electron beam melting technology (EBM) allows the fabrication of customized implants with tailored mechanical properties and high potential in the clinical practice. In order to augment the interaction with the biological tissue, stem cells have recently been combined with metallic scaffolds for skeletal engineering applications. We previously demonstrated that human embryonic stem cell-derived mesodermal progenitors (hES-MPs) hold a great potential to provide a homogeneous and unlimited supply of cells for bone engineering applications. This study demonstrates the effect of EBM-fabricated cp-Ti and Ti6Al4V porous scaffolds on hES-MPs behavior, in terms of cell attachment, growth and osteogenic differentiation. Displaying different chemical composition but similar surface properties, EBM-fabricated cp-Ti and Ti6Al4V scaffolds supported cell attachment and growth, and did not seem to alter the expression of genes involved in osteogenic differentiation and affect the alkaline phosphatase activity. In conclusion, interfacing hES-MPs to EBM-fabricated scaffolds may represent an interesting strategy for design of third-generation biomaterials, with the potential to promote implant integration in clinical conditions characterized by poor bone quality.

## 1. Introduction

Bone engineering aims at fabricating bone substitutes for the reconstruction of skeletal defects caused by degenerative disorders and trauma [1]. Hard materials, such as metals and their alloys, have been extensively used as load-bearing and reconstructive implants for skeletal engineering applications [2]. cp-Ti and the Ti6Al4V alloy are mainly employed due to their biocompatibility and good mechanical properties [3], as well as the ability to become osseointegrated by forming a direct contact with the surrounding bone [4–6]. Moreover, both the cp-Ti and Ti6Al4V alloy form a passive oxide layer at the surface, which imparts resistance to corrosion after implantation in the human body [7]. However, when in contact with physiological solutions both cp-Ti and Ti6Al4V tend to release metal ions, raising concerns about the possible cytotoxic effects associated with leaching of vanadium from the Ti6Al4V alloy [8, 9].

In many cases, a complex three-dimensional (3D) geometry of the implantable device is needed for an optimal clinical outcome [10]. Furthermore, a certain degree of porosity may facilitate bone ingrowth and remodeling throughout the implant, eventually enhancing bone-material contact and stability. Implant porosity also allows reducing stiffness mismatch between bone and the implanted material, hence helps to avoid stress-shielding effects [11]. Complex porous 3D metallic parts are currently manufactured using innovative free-form fabrication (FFF) techniques, such as 3D printing [12], sacrificial wax template [13], 3D fiber deposition technique [14], selective laser melting [15], selective laser sintering [16], direct metal deposition [17], and electron beam melting (EBM) [18–20]. Among these, EBM represents a promising technique for the high-speed and high-volume fabrication of customized metallic implants with excellent properties for personalized applications in skeletal engineering [20]. 

The possibility to interface stem cells to implants before implantation has been explored in order to augment bone ingrowth and promote osseointegration [21]. Human mesenchymal stem cells (hMSCs), which reside in the bone marrow [22] and other adult tissues [23–26], have been largely used for tissue engineering applications and recently combined with metallic scaffolds for bone engineering applications [27–30]. However, hMSCs manifest important limitations for the large-scale production of cells for clinical applications, especially considering harvesting, isolation, and enrichment procedures, which result in a high degree of heterogeneity [31, 32], as well as the limited proliferation potential and the loss of functionality observed after protracted expansion [33–35]. An alternative to hMSCs is the use of human stem cell-derived mesodermal progenitors (hES-MPs), which do not form teratoma and resemble hMSCs in terms of gene expression and lineage commitment but display higher regeneration potential [35–37], which is fundamental for the bulk production of functional cells for engineering applications. However, for a potential clinical use of hES-MPs in skeletal engineering applications, the understanding of their behavior when interfaced with 3D metallic scaffolds *in vitro* is crucial, but no information about this is available today. 

In the present study, we interfaced hES-MPs with EBM-fabricated 3D cp-Ti and Ti6Al4V porous scaffolds, with the aim of investigating the effect of these materials in influencing the hES-MPs ability to attach, grow, and differentiate toward the osteogenic lineage.

## 2. Material and Methods

### 2.1. Free-Form Fabrication

**Figure 1:**
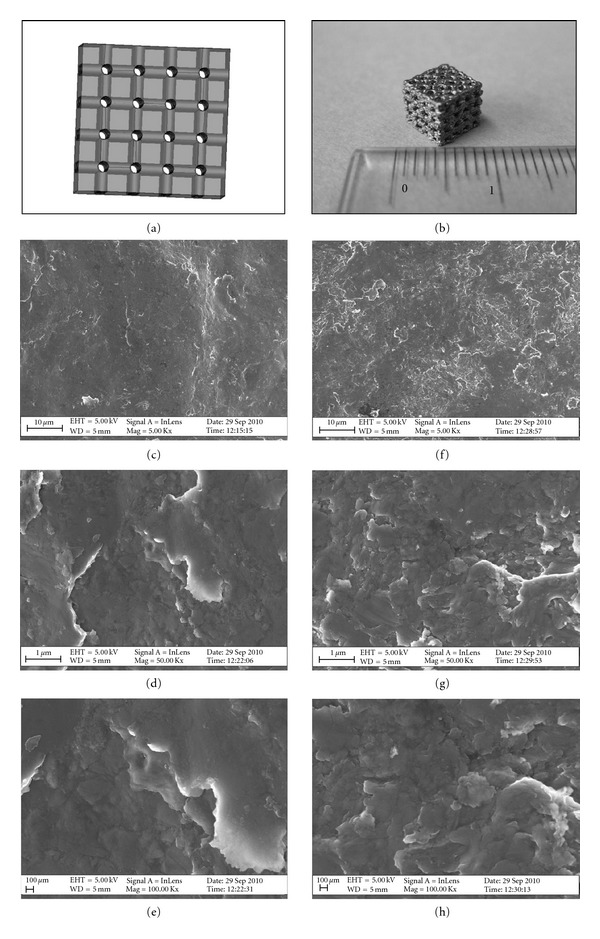
(a) CAD model used for the EBM-fabrication of cp-Ti and Ti6Al4V scaffolds. (b) A photograph showing the scaffolds used in the present study. (c–h) SEM images showing the surface topography of cp-Ti (c–e) and Ti6Al4V (f–h) scaffolds at increasing magnifications; scale bar = 10 *μ*m, 1 *μ*m, and 100 nm, respectively.

The free-form-fabricated scaffolds were produced in an Arcam EBM S12 system (Arcam AB, Mölndal, Sweden; http://www.arcam.com/) from standard Arcam cp-Ti and Ti6Al4V extra low interstitial powders with a particle size of 45–100 *μ*m. The cubic scaffolds were made from the 3D computer-aided design (CAD) model shown in Figure 1(a), with each side measuring approximately 6.8 mm. In Figure 1(b), a photograph of the EBM-fabricated scaffolds is shown. The electron beam melts the powder in a layer-by-layer process with a layer thickness of 0.1 mm. All the scaffolds were produced in two separate builds, one for Ti6Al4V and one for cp-Ti. The building temperature of the powder bed was 750°C for Ti6Al4V and 700°C for cp-Ti, respectively. The vacuum pressure inside the chamber was 2 × 10^−3^ mbar for both builds. The control settings of the build process were in accordance with standard settings provided by Arcam AB. After building, the scaffolds were cooled in an He environment at a pressure of 2 × 10^2^ mbar until they reached a temperature of about 100°C. Air was then introduced into the chamber. All scaffolds were finally blasted with the same cp-Ti and Ti6Al4V ELI powders they were built of. Both the cp-Ti and Ti6Al4V had an average pore size of 620 *μ*m in diameter and a volume porosity of about 70–75%. All pores were interconnected [38].

### 2.2. Cleaning and Sterilization

After blasting, the scaffolds were ultrasonically treated in order to remove impurities. First they were ultrasonically cleaned for 5 minutes each in two successive baths of MIS 024 (Tremedic AB, Göteborg, Sweden), then three times for 5 min in ELGA/Milli-Q water (Tremedic AB). After this, the scaffolds were let to dry in air in a sterile environment. The scaffolds were then sealed in sterile bags and steam autoclaved for 15 min at 120°C.

### 2.3. Surface Analysis of Scaffolds

Surface topography of native scaffolds was investigated by scanning electron microscopy (SEM), using a Leo Ultra 55 FEG SEM (Leo Electron Microscopy Ltd., Cambridge, UK) equipped with a secondary electron detector and an in-lens detector. Overview images were acquired in the secondary electron imaging mode at 5 kV acceleration voltage. The in-lens detector was used for closer examination of the samples at 2 kV acceleration voltage.

Surface composition and oxide thickness was analyzed by time-of-flight secondary ion mass spectroscopy (TOF-SIMS) using a TOF-SIMS IV instrument (ION-TOF GmbH, Münster, Germany) equipped with bismuth and C_60_ cluster ion sources. Before analysis, samples were ultrasonicated in ethanol (99%) for 5 min (3 times), followed by ELGA/Milli-Q water (Tremedic AB) for 15 minutes. Then, samples were dried in a flow of N_2_ gas before analysis. Mass spectra of positive and negative secondary ions were measured from two areas of 100 × 100 *μ*m^2^ (divided into 128 pixels)^2^ on each sample, with a data acquisition time of 50 s. Bi_3_^+^ (25 keV) was used as primary ions, with a target current of 0.1 pA. Each spectrum was calibrated by assigning theoretical masses to known peaks. For depth profiles, the sample areas were sputtered with a beam of C_60_^+^ ions (10 keV, target current 0.4-0.5 nA, area 250 × 250 *μ*m^2^). The depth profiles (positive mode) were done with cycles of sputtering for 5 s, followed by data acquisition for 10 s (same area and pixels as above) with a pause of 1 s. The depth profile measurements were continued until the TiO_2_-related peak (main TiO^+^ at m/z 64) intensity had leveled out in the metal (after 250–300 s of total C_60_^+^ sputtering). The thickness of the oxide layer measured in the depth profiles was calculated from the time required to sputter through the oxide layer. Conversion of the sputter time to depth was done by calibration of the sputter rate of TiO_2_ on a standard titanium plate with a known oxide thickness.

### 2.4. Cell Derivation and Expansion

hES-MPs were provided by Cellartis (Gothenburg, SE; http://www.cellartis.com/). The hES-MPs were derived from an undifferentiated hES cell line (SA002.5), and their derivation and characterization has been described earlier [36]. Briefly, undifferentiated hES cells were removed from the supporting feeder layer and plated onto 0.1% porcine gelatin-coated cell culture dishes (BD Falcon/BD Biosciences, Bedford, MA, USA) in medium consisting of DMEM-HG (PAA Laboratories, Linz, Austria) supplemented with 1% Penicillin-Streptomycin (PEST, PAA Laboratories), L-glutamine (2 mM, Gibco, Paisley, UK), 10% fetal bovine serum (FBS, Gibco), and 10 ng/mL bFGF (Invitrogen, Paisley, United Kingdom). To initiate the derivation of hES-MPs, the hESCs were enzymatically passaged as a single cell suspension using TrypLE Select (Invitrogen) and were subsequently plated onto gelatin-coated culture dishes. This procedure was repeated every 7 days until the cell population became homogeneous for a mesenchymal morphology. Following the initial derivation steps, the hES-MPs were cultured in uncoated tissue culture flasks (BD Falcon/BD Biosciences) in a humidified atmosphere at 37°C and 5% CO_2_ and enzymatically passaged with TrypLE Select every 7 days. 

Cells were expanded in the medium described above. Medium was changed every 3-4 days and cells were passaged when reaching 80% confluence. Cells were cultured at 37°C in 5% CO_2_.

### 2.5. Cell Seeding

A centrifugal seeding technique was used in order to increase cell penetration and attachment across the scaffolds [39]. Scaffolds were placed in round-bottom tube, and 1 mL of cell suspension (10^6^ cell/mL) was added per each scaffold. Tubes containing the cell-scaffold constructs were then spun at 1200 rpm for 5 min. After centrifugation, unattached cells were resuspended and seeded again. The centrifugation procedure was repeated 5 times. Cell/scaffold constructs were finally incubated at 37°C in 5% CO_2_ for 24 h.

### 2.6. Osteogenic Stimulation

hES-MPs were cultured under osteogenic conditions for 6 weeks. Briefly, cell/scaffold constructs were cultured in 15 mL tubes (Falcon, BD, Franklin Lakes, NJ, USA) in DMEM-LG (PAA Laboratories) supplemented with 1% PEST, L-glutamine (2 mM), 10% FBS, L-ascorbic acid (4.5 × 10^−5^ M; Sigma-Aldrich, St. Louis, MO, USA), dexamethasone (10^−6^ M; Sigma-Aldrich), and *β*-glycerophosphate (2 × 10^−2^ M; Calbiochem, Darmstadt, Germany). Cell/scaffold constructs were incubated at 37°C in 5% CO_2_. Medium was changed twice a week.

### 2.7. Cell Attachment and Distribution

Cell attachment across the scaffolds was investigated by scanning electron microscopy (SEM) 2 days after culture under osteogenic conditions. For SEM investigation, cell/scaffold constructs were rinsed twice in phosphate saline buffer (PBS) before adding them in a modified solution of Karnovsky fixative, consisting of a solution of sodium azide (0.02%; Fluka Biochemika GmbH, Buchs, Switzerland), paraformaldehyde (2%; VWR International, Stockholm, Sweden) and glutaraldehyde (2.5%; Agar Scientific, Stansted, England) in sodium cacodylate buffer (0.05 M; Agar Scientific). Then, cell/scaffold constructs were treated with a solution of OsO_4_ (1%; Agar Scientific) in sodium-cacodylate buffer and stored at 4°C for 4 h. After rinsing 5 times with distilled H_2_O, samples were treated with hexamethyldisilazane (1%; Fluka, Sigma) before adding again a solution of OsO_4_ (1%) in sodium cacodylate buffer (0.1 M). After dehydration with ethanol of increasing concentration, samples were treated twice (10 min) with a solution of hexamethyldisilazane (1%; Fluka, Sigma) and dried overnight. For analysis, samples were sputter coated (EMITECH K550X; EMITECH, Kent, UK) with palladium for 2 min at 25 mA before SEM examination. The SEM analysis was performed as described above.

### 2.8. DNA Content

In order to compare cell proliferation across the cp-Ti a and Ti6Al4V scaffolds, the total DNA content was measured after 1 and 2 weeks of culture under osteogenic conditions. Briefly, samples were rinsed twice with PBS and then treated with a solution of papain (Sigma-Aldrich) for about 5 min. Samples were then placed in oven at 60°C for 1 h for homogenization. Afterwards, lysates were collected, placed in Eppendorf tubes, and left in oven at 60°C overnight to allow drying. Samples were later dissolved in 200 *μ*L of phosphate buffer containing EDTA (PBE; 5 mM, pH 7.5) and stained with a solution of Hoechst 33258 (3.7 × 10^−8^ M; Sigma-Aldrich) in PBE buffer. After an incubation period of 10 min in the dark, samples were excited by ultraviolet light at 360 nm and emission read at 460 nm in a SPECTRAmax GEMININI (Medical Devices, Inc., Sunnyvale, CA, USA) microplate reader using the Softmax Pro software (Medical Devices, Inc.). A standard curve consisting of serially diluted calf thymus DNA was run in the same microplate.

### 2.9. Flow Cytometry Analysis

In order to estimate the rate of cellular death for cells seeded both onto cp-Ti and Ti6Al4V, samples were harvested after 5 days of culture under osteogenic conditions and cells analyzed using the FITC Annexin V Apoptosis Detection Kit I (Becton Dickinson, Franklin Lakes, NJ, USA). Briefly, cells were trypsinated for about 10 min, washed twice with PBS, and resuspended in 1X binding buffer (Becton Dickinson) before staining. Cells were stained with 5 *μ*L of FITC Annexin V and 5 *μ*L of propidium iodide (PI) and incubated for 15 min at room temperature in the dark. Cells were analyzed by flow cytometry within 1 h. All samples were analyzed using the FACS Aria flow cytometer (Becton Dickinson) using the FACS Diva software (Becton Dickinson).

### 2.10. Lactate Dehydrogenase Activity

In order to investigate the proportion of cellular death, the activity of lactate dehydrogenase (LDH) released into the cell culture medium was measured weekly. Briefly, medium was collected and the LDH activity was analyzed using a Cytotoxicity Detection Kit. The LDH activity was determined in a coupled enzymatic reaction during which NAD^+^ is reduced to NADH. The formation rate of NADH was measured at 340 nm and considered proportional to the catalytic activity of LDH. 

The analysis was performed at the accredited laboratory of Sahlgrenska University Hospital.

### 2.11. Quantitative Real-Rime PCR

In order to examine osteogenic differentiation, cells seeded onto cp-Ti and Ti6Al4V were analyzed by real-time (RT) PCR after 1 and 2 weeks of culture under osteogenic conditions. Cell-scaffold constructs were treated with a solution of RLT buffer, with addition of 1%  *β*-mercaptoethanol, and the lysate vortexed for one min. The lysate was later transferred directly into a QIAshredder spin column and centrifuged for 2 min. Total RNA from the samples was extracted using RNeasy Minikit (QIAGEN GmbH, Hilden, Germany) according to the manufacturer's instructions. DNAse treatment was performed in order to eliminate any contamination from genomic DNA. Reverse transcription was carried out using iScript cDNA Synthesis Kit (Bio-Rad, Hercules, USA) in a 10 *μ*L reaction, according to the manufacturer's instructions. Design of primers for *RUNX2, COL1A1, OPN*, and* OC* was performed using the Primer3 web-based software [40]. Design parameters were adjusted to minimize formation of artifact products and to be able to use an annealing temperature in the PCR at about 60°C. Primers were designed to yield short amplicons (preferably shorter than 200 bp) and to function well with SYBR Green I fluorescent dye for detection of the PCR products in real time. Primer sequences are available at TATAA Biocenter AB (http://www.tataa.com).

RT-PCR was performed in duplicates using the Mastercycler ep realplex (Eppendorf, Hamburg, Germany) in 20 *μ*L reactions. Cycling conditions were 95°C for 10 min followed by 45 cycles of 95°C for 20 s, 60°C for 20 s, and 72°C for 20 s. The fluorescence was read at the end of the 72°C step. Melting curves were recorded after the run by stepwise temperature increase (1°C/5 s) from 65 to 95°C.

Quantities of target genes were presented as normalized to the number of cell using the expression of 18S ribosomal subunit. Normalized relative quantities were calculated using the delta Ct method and 90% PCR efficiency (k*1.9^∆ct^).

### 2.12. Alkaline Phosphatase Activity

After 10 days of culture under osteogenic conditions, the alkaline phosphatase (ALP) activity was measured following the lysis of the cells using M-PER (Fisher Scientific, Gothenburg, Sweden). ALP activity was assayed by using p-nitrophenylphosphate as substrate. The quantity (in alkaline solution) of p-nitrophenol produced, which exhibits an absorbance maximum at 405 nm, was considered directly proportional to the alkaline phosphatase activity. The analysis was performed at the accredited laboratory of Sahlgrenska University Hospital.

### 2.13. Histology

After 6 weeks of culture cell-scaffold, constructs were plastic embedded and ground sectioned for histological analysis. Briefly, cp-Ti and Ti6Al4V constructs were rinsed twice with PBS and fixated in 4% formaldehyde for 4 h at 3–5°C. After fixation, constructs were rinsed three times with ELGA/Milli-Q water (Tremedic AB) on an orbital shaker. After dehydration with ethanol of increasing concentration, the constructs were embedded in plastic resin (LR White, the London Resin Co. Ltd., Hampshire, UK) and hardened on ice. Samples were cut using the Exakt cutting-Grinding equipment as described elsewhere [41]. The final section was approximately 50–60 *μ*m thick. The scaffolds with surrounding cells were stained with toluidine blue staining before cover glass mounting, and observed using light microscopy (Eclipse E600; Nikon, Japan).

### 2.14. Statistical Analysis

Results are expressed as means and standard deviations. Three samples were used per each analysis. Differences were determined by the nonparametric Mann-Whitney test for independent samples using the SPSS Statistics 17.0 software. For all the analyses, a value of *P* ≤ 0.05 was considered as significant difference.

## 3. Results

### 3.1. Topography and Surface Chemical Composition

In Figure 1, SEM images showing the topography of cp-Ti (c–e) and Ti6Al4V (f–h) scaffolds at different magnification are shown. While slight differences were observed in surface roughness at the micron scale level between scaffolds of cp-Ti (Figures 1(c) and 1(d)) and Ti6Al4V (Figures 1(f) and 1(g)), both materials displayed a similar topography, with relatively flat areas and areas with flakes and protrusions as seen in the high-magnification SEM images displayed in Figures 1(e) and 1(h), respectively. 

**Figure 2:**
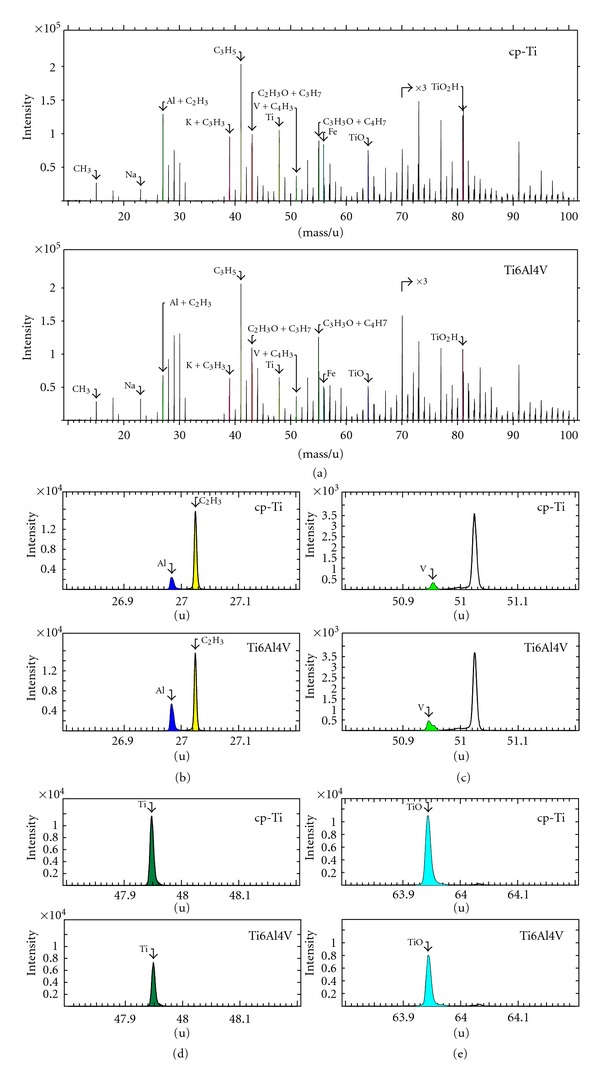
(a) TOF-SIMS spectra showing the elemental surface compositions of the cp-Ti and Ti6Al4V scaffolds. Enlargements of the spectra displaying the Al^+^ (b), V^+^ (c), Ti^+^ (d), and TiO^+^ (e) peaks.

ToF-SIMS spectra from the surface of the cp-Ti and Ti6Al4V samples were similar (Figure 2(a)), indicating no major differences in surface chemical composition between the two materials. The spectra were dominated by organic fragment ions of the type C_x_H_y_^+^ and C_x_H_y_O^+^ (presumably due to adsorbed organic molecules from the ambient) and signals that can be attributed to TiO_2_ (e.g., Ti^+^, TiO^+^, Ti_2_O^+^, Ti_2_O_3_^+^). However, the alloy-related ion peaks, specifically Al (blue) and V (green), were higher in the Ti6Al4V samples compared to the cp-Ti samples as shown in Figures 2(b) and 2(c), respectively. On the other hand, titanium-related peaks were slightly higher in the cp-Ti samples than the alloyed samples as shown in Figures 2(d) and 2(e). Spectra measured after removal of the oxide layer were dominated by Ti signals. In addition, the alloys showed clear signals from the alloy elements Al and V. 

TOF-SIMS analysis of the scaffold surface showed no significant differences in oxide layer thickness between the cp-Ti and Ti6Al4V scaffolds, resulting in an average thickness of 24 nm for both cp-Ti and Ti6Al4V scaffolds (Table 1). 

### 3.2. Cell Attachment

**Figure 3:**
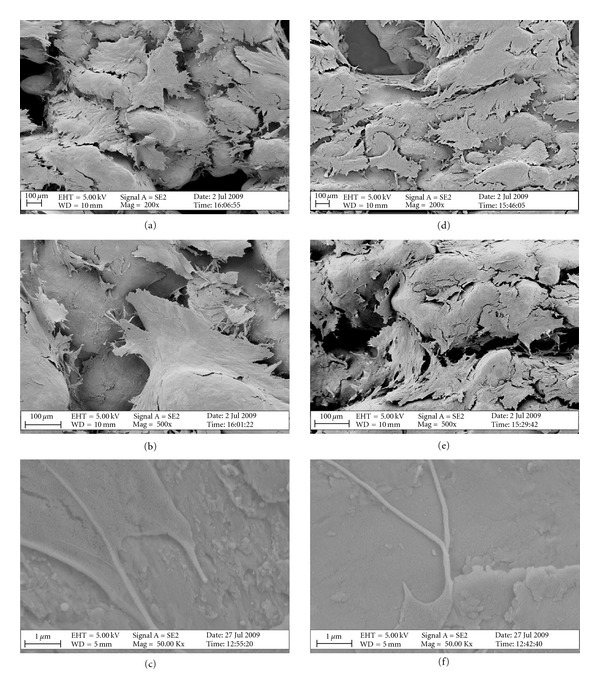
SEM images showing hES-MPs morphology and distribution across the cp-Ti (a–d) and Ti6Al4V (e–h) scaffolds; scale bar = 200 *μ*m (a, b, d, and f) and 1 *μ*m (c and f).

Cell attachment and distribution across the scaffolds were assessed by SEM investigation 2 days after seeding.  Figure 3 demonstrates that both cp-Ti (Figures 3(a)–3(c)) and Ti6Al4V (Figures 3(d)–3(f)) constructs displayed a homogenous distribution of cells across the scaffold (Figures 3(a) and 3(b) resp.), with a dense layer of cells covering the entire surface. Cells displayed a flat morphology with cellular protrusions extending from the cellular periphery both on cp-Ti (Figures 3(a) and 3(b)) and Ti6Al4V (Figures 3(d) and 3(e)) scaffolds. In proximity of the scaffold pores, cells appeared to follow the curvature of the surface and stretch out inside the pore both when seeded on cp-Ti (Figure 3(b)) and Ti6Al4V (Figure 3(e)) scaffolds. Figures 3(c) and 3(f) show details of cellular filopodia interacting with the underlying surface for cp-Ti and Ti6Al4V scaffolds, respectively.

### 3.3. Cell Proliferation and Viability

**Figure 4:**
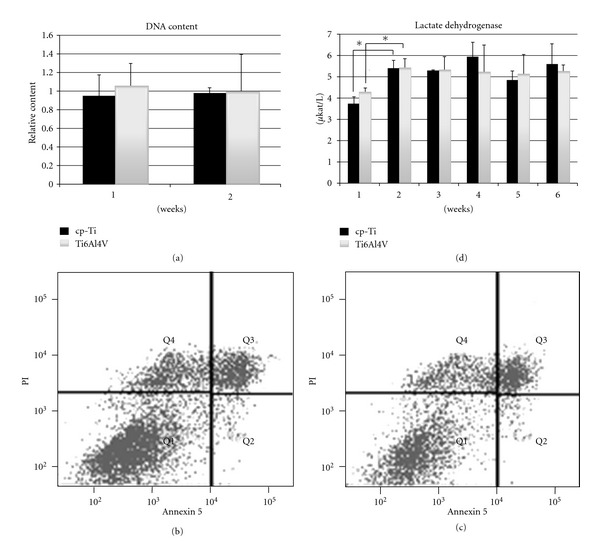
(a) Histograms showing the content of DNA for both constructs after 1 and 2 weeks of culture. (b-c) Flow cytometry plots displaying the proportion of viable (Q1), apoptotic (Q2, Q3), and dead (Q4) cells are shown for cp-Ti (b) and Ti6Al4V (c) constructs. (d) Histograms showing the LDH activity for both constructs along the entire duration of the experiment. A value of *P* < 0.05 was taken as a significant difference (*).

The effect of cp-Ti and Ti6Al4V scaffolds on cell proliferation was investigated by measuring the DNA content after 1 and 2 weeks of culture. In Figure 4(a), the DNA content for cp-Ti and Ti6Al4V constructs is shown. No significant differences were observed between the two constructs after 1 and 2 weeks of culture. 

Cell viability was assessed by flow cytometry after 1 week of culture and by measuring the LDH activity weekly along the entire duration of the experiment. In Figures 4(b) and 4(c), the proportion of viable (Q1), apoptotic (Q2, Q3), and dead (Q4) cells is shown for cp-Ti and Ti6Al4V constructs, respectively. For both scaffolds, a similar pattern was observed, with 69% and 71% of the total counted cells found to be viable for cp-Ti and Ti6Al4V scaffolds, respectively. LDH results displayed a similar pattern for both constructs as shown in Figure 4(d). After a significant increase in LDH activity detected after 2 weeks, no significant variations were found for the following time points investigated and between constructs of cp-Ti and Ti6Al4V.

### 3.4. Histology

Histological investigation was used to assess cell growth across the scaffolds. In Figure 7, toluidine-stained sections for both constructs of cp-Ti (Figures 7(a)–7(c)) and Ti6Al4V (Figures 7(d)-7(e)) are shown. hES-MPs grew uniformly on both scaffolds and formed cellular layers following the irregularities of the underlying material surface.

### 3.5. Osteogenic Differentiation

**Figure 5:**
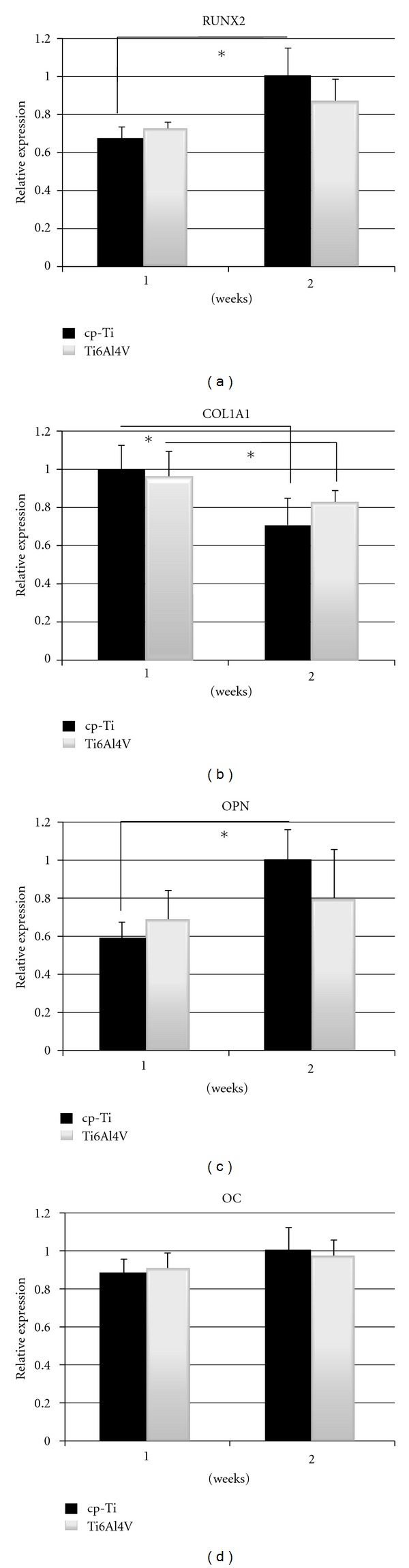
Real-time PCR results showing the expression of *RUNX2*, *COL1A1*, *OPN*, and *OC *after 1 and 2 weeks of culture for both constructs. Results are presented as relative expression values. A value of *P* < 0.05 was taken as significant difference (*).

**Figure 6:**
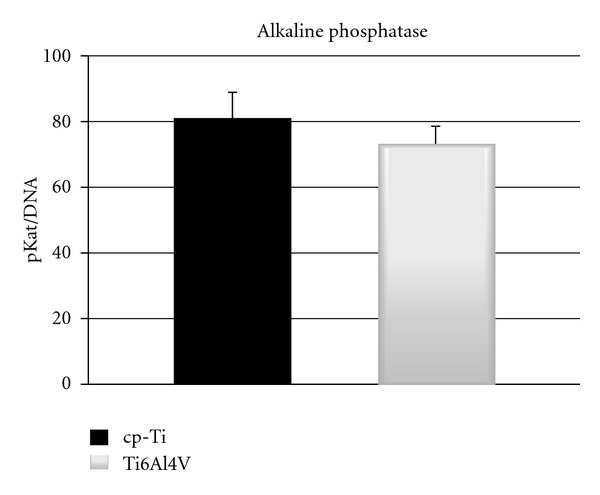
Histograms showing the ALP activity for both constructs after 10 days of culture.

**Figure 7:**
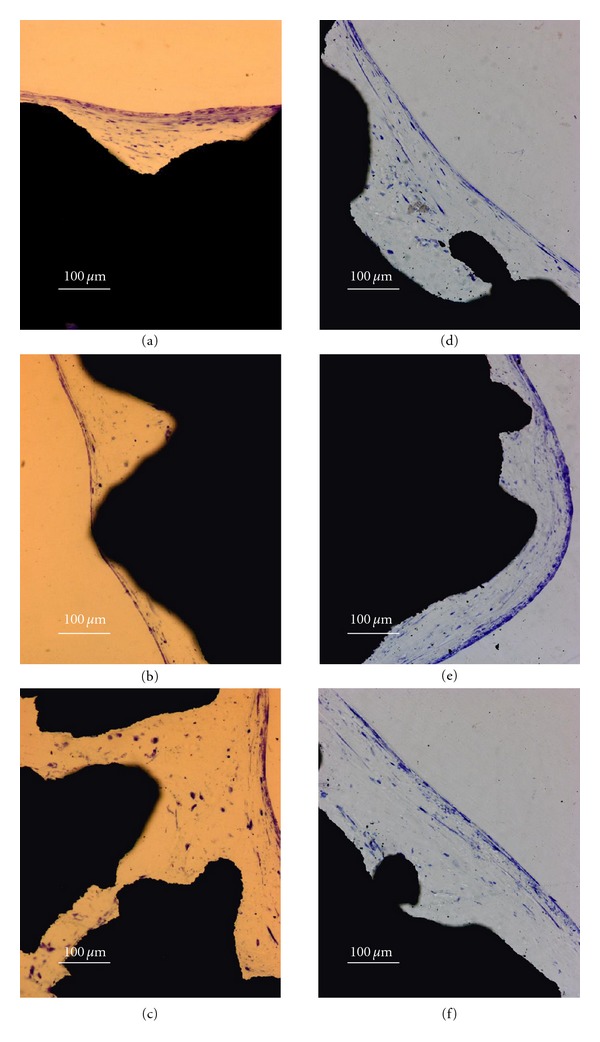
Histological micrographs of cp-Ti (a–c) and Ti6Al4V (d–f) constructs stained with toluidine blue; scale bar = 100 *μ*m.

The effect of material surface composition on osteogenic differentiation was investigated by real-time PCR and ALP activity measurements. In Figure 5, the expression of *RUNX2*, *COL1A1*, *OPN*, and *OC* is shown. Per each gene the higher expression value was used as baseline. Both constructs of cp-Ti and Ti6Al4V displayed increased expression of *RUNX2* after 2 week of culture (Figure 5(a)), although the observed increase was found to be significant only for constructs of cp-Ti. No differences were observed between the two types of constructs. On the other hand, *COL1A1* displayed a significant decrease in expression after 2 weeks for both constructs (Figure 5(b)), but no differences were observed when hES-MPs were seeded on cp-Ti or Ti6Al4V scaffolds.  Figure 5(c) shows that the expression of *OPN* displays an increase after 2 weeks for both constructs. However, the observed increase was found to be significant only for cp-Ti constructs. No variations were observed in the expression of* OC* between weeks 1 and 2, and between constructs of cp-Ti and Ti6Al4V (Figure 5(d)).  Figure 6 shows the ALP activity data after 10 days of culture. The data demonstrated similar activity for both constructs of cp-Ti and Ti6Al4V.

## 4. Discussion

Free-form fabrication of implantable devices is emerging as an attractive technique for the production of 3D complex biomaterials for personalized applications in skeletal engineering. EBM has recently been used to fabricate cp-Ti and Ti6Al4V implants with excellent material properties [19, 42, 43] and has the ability to provide geometrical features, which support bone ingrowth and ensure a stable osseointegration [20]. By varying the size of the pores and struts, EBM allows the modulation of device porosity and tailoring of optimal mechanical properties to meet specific requirements in different applications. An interesting concept is to interface biomaterials with stem cells in order to speed up the regeneration process and/or achieve better osseointegration of the implants, especially in situations characterized by poor bone quality or otherwise compromised regenerative capacity [44]. hMSCs are experimentally relevant cells but manifest important limitations for the large-scale production of cells, especially considering the limited proliferative potential and loss of functionality following *in vitro* expansion [33, 35, 37] or aging. On the other hand, hES-MPs are able to provide an unlimited source of functional osteoprogenitor cells for clinical applications.

The present study demonstrates that hES-MPs can be interfaced with EBM-fabricated cp-Ti and Ti6Al4V under *in vitro* conditions and may be successfully used in the design of third-generation biomaterials for skeletal engineering applications. Soon after seeding, hES-MPs formed a dense layer of cells in tight contact with the geometrical features of the cp-Ti and Ti6Al4V scaffolds, both in an orientation parallel and perpendicular to the built-in direction. When studying cell proliferation, no significant overtime increase in the content of DNA was detected for both constructs of cp-Ti and Ti6Al4V. It is recognized that increasing cell density arrests cell proliferation via the process of contact inhibition [45], and cell-cell interaction and communication are essential events for tissue formation [46]. Moreover, upon cell differentiation, the expression of genes involved in proliferation has been reported to be downregulated, indicating that a proliferation/differentiation switch exists [47]. In this perspective, especially considering the high proliferative potential of hES-MPs [35], the formation of a dense layer of cells, as demonstrated by SEM investigation after 2 days of culture, it is likely to explain the DNA content results observed. A similar proliferation/differentiation switch occurring at an early stage of *ex vivo* culture may represent an advantageous condition for a clinical use of the cell/scaffold constructs used in this study. On the other hand, after an initial period of high proliferative state, a stable balance between cell proliferation and cell death may have been reached, as previously observed in our laboratory. For example, flow cytometry data reported in the present study resulted in a cellular live/dead ratio of about 70/30 after the first week of culture for both constructs of cp-Ti and Ti6Al4V, indicating that under osteogenic condtions cell death is an ongoing process, with possible biological importance during cell differentiation. In this regard, Lynch et al. reported evidence that cells undergo programmed cell death upon osteogenic differentiation *in vitro* [48]. However, when studying the LDH activity, a significant increase was observed between the first and second weeks of culture for both constructs of cp-Ti and Ti6Al4V, which possibly reflects an increased number of total cells and, as consequence, an increased number of cells undergoing cell death. The observed results are not in accordance with DNA content data suggesting that, although cell proliferation was ongoing between the first and second weeks of culture, technical limitations in DNA extraction may account for the discrepancy observed between the DNA content and LDH activity results. In a different fashion, cell viability may decrease over time, as reported by Muller et al. after seeding SAOS-2 cells on cp-Ti porous scaffolds [49]. Interestingly, the LDH activity data showed that, after the first 2 weeks, the cellular live/dead ratio became stable until the sixth week of culture and was not influenced by the type of material used. The finding excludes any short-term cytotoxic effect associated with the presence of vanadium within the Ti6Al4V alloy. The histological analysis corroborates these other results, showing similar pattern of cells surrounding both constructs of cp-Ti and Ti6Al4V. Taken together, the above data suggests a similar influence of both materials on hES-MPs. However, it is clear that additional studies, both *in vitro* and *in vivo*, are fundamental to fully assess the effect of the investigated materials on hES-MPs. 

At the implant site, circulating progenitor cells migrate, adhere and differentiate into functional osteoblasts, eventually favoring bone tissue formation and healing [24]. In this view, it is important to investigate whether the materials used in this study influence the expression of genes involved in osteogenic differentiation. In our study, the expression level of *RUNX2*, which is a transcriptional activator of osteoblastic differentiation [50], displayed an increase after 2 weeks of culture for both cp-Ti and Ti6Al4V scaffolds. The observed profiles do not correspond to those observed during our previous 2-dimensional studies, in which hES-MPs were characterized by a general decrease in *RUNX2* expression at week 2, indicating that cells may respond differently when cultured under 3D conditions [51]. On the other hand, the material composition and topography of the scaffolds used may play a synergistic effect in affecting gene expression and promoting osteogenic differentiation. A similar trend was observed for genes whose expression is recognized to be under the control of *RUNX2* [52], such as *OPN* and *OC*, further supporting the idea that both scaffolds used in the present study possess osteoconductive properties [53]. On the contrary, *COL1A1* displayed a significant decrease in expression for both constructs of cp-Ti and Ti6Al4V, highlighting the complex biology underlying gene expression, as well as the possibility that additional components control its regulation. In this regard, Tadic et al. demonstrated that Dlx5 played a role in regulating the expression of *COL1A1* into chick calvarial periosteal cells [54]. A similar decrease in *COL1A1* expression was reported by Bedi et al. after culturing human fetal osteoblast on Ti6Al4V scaffolds [55]. Interestingly, for all genes investigated, the expression profile observed was similar when hES-MPs were interfaced to cp-Ti and Ti6Al4V. In a similar fashion, when studying the activity of ALP, which is recognized to play a central role in osteogenesis and mineralization of the extracellular matrix [56, 57], no differences were observed between the two scaffold materials.

Altogether, the data reported in this study demonstrate the potential of hES-MPs to be interfaced to EBM-fabricated scaffolds. Displaying different chemical composition but similar surface properties with respect to topography, oxide thickness and composition, EBM-fabricated cp-Ti and Ti6Al4V scaffolds supported cell attachment and growth, and did not seem to alter the expression of genes involved in osteogenic differentiation and affect the activity of ALP. The materials did not exhibit any major adverse effect on hES-MPs behavior. Based on this observation, interfacing hES-MPs to EBM-fabricated scaffolds might represent an interesting alternative in the design of third-generation biomaterials for skeletal engineering applications. However, *in vivo* studies are fundamental to evaluate the true potential of hES-MPs to drive bone formation and promote implant osseointegration in clinical situations.

## 5. Conclusions

The ability to fabricate 3D metallic parts with complex geometrical features and tailored mechanical properties opens the possibility to design customized biomaterials that, in combination with patient-specific hES-MPs, hold the potential to promote bone formation and improve implant integration in clinical conditions characterized by poor bone quality.

##  Acknowledgments 

The authors sincerely thank Birgitta Norlindh for helping with histology. They acknowledge BIOMATCELL VINN Excellence Center of Biomaterials and Cell Therapy, Region Västra Götaland, Swedish Research Council (K2009-52X-09495-22-3 and 2005–7544), and JOIN (ed) T Marie Curie Action for the financial support of the study.

## Figures and Tables

**Table 1:** The calculated oxide thicknesses for the cp-Ti and Ti6Al4V scaffolds, showing the mean value and standard deviation.

Oxide thickness	
Material	Th (nm)
cp-Ti 1	26.3
cp-Ti 2	24.2
cp-Ti 3	23.0
cp-Ti 4	24.1

AVERAGE	24.4

STDEV	1.4

Ti6Al4V 1	30.0
Ti6Al4V 2	22.1
Ti6Al4V 3	23.7
Ti6Al4V 4	21.8

AVERAGE	24.4

STDEV	3.8
